# Comparison of protein coding gene contents of the fungal phyla Pezizomycotina and Saccharomycotina

**DOI:** 10.1186/1471-2164-8-325

**Published:** 2007-09-17

**Authors:** Mikko Arvas, Teemu Kivioja, Alex Mitchell, Markku Saloheimo, David Ussery, Merja Penttila, Stephen Oliver

**Affiliations:** 1 VTT, Tietotie 2, Espoo, P.O. Box 1500, 02044 VTT, Finland; 2 Biomedicum, P.O. Box 63 (Haartmaninkatu 8), FI-00014 University of Helsinki, Finland; 3 EMBL Outstation – Hinxton, European Bioinformatics Institute, Welcome Trust Genome Campus, Hinxton, Cambridge, CB10 1SD, UK; 4 Center for Biological Sequence Analysis BioCentrum-DTU The Technical University of Denmark DK-2800 Kgs. Lyngby, Denmark; 5 University of Manchester, Michael Smith Building, Oxford Road, Manchester, M13 9PT, UK

## Abstract

**Background:**

Several dozen fungi encompassing traditional model organisms, industrial production organisms and human and plant pathogens have been sequenced recently and their particular genomic features analysed in detail. In addition comparative genomics has been used to analyse specific sub groups of fungi. Notably, analysis of the phylum Saccharomycotina has revealed major events of evolution such as the recent genome duplication and subsequent gene loss. However, little has been done to gain a comprehensive comparative view to the fungal kingdom. We have carried out a computational genome wide comparison of protein coding gene content of Saccharomycotina and Pezizomycotina, which include industrially important yeasts and filamentous fungi, respectively.

**Results:**

Our analysis shows that based on genome redundancy, the traditional model organisms *Saccharomyces cerevisiae *and *Neurospora crassa *are exceptional among fungi. This can be explained by the recent genome duplication in *S. cerevisiae *and the repeat induced point mutation mechanism in *N. crassa*.

Interestingly in Pezizomycotina a subset of protein families related to plant biomass degradation and secondary metabolism are the only ones showing signs of recent expansion. In addition, Pezizomycotina have a wealth of phylum specific poorly characterised genes with a wide variety of predicted functions. These genes are well conserved in Pezizomycotina, but show no signs of recent expansion. The genes found in all fungi except Saccharomycotina are slightly better characterised and predicted to encode mainly enzymes. The genes specific to Saccharomycotina are enriched in transcription and mitochondrion related functions. Especially mitochondrial ribosomal proteins seem to have diverged from those of Pezizomycotina. In addition, we highlight several individual gene families with interesting phylogenetic distributions.

**Conclusion:**

Our analysis predicts that all Pezizomycotina unlike Saccharomycotina can potentially produce a wide variety of secondary metabolites and secreted enzymes and that the responsible gene families are likely to evolve fast. Both types of fungal products can be of commercial value, or on the other hand cause harm to humans. In addition, a great number of novel predicted and known enzymes are found from all fungi except Saccharomycotina. Therefore further studies and exploitation of fungal metabolism appears very promising.

## Background

At least 36 fungal genomes are already available at public databases and several more are being sequenced. Of eukaryotes, Fungi is the kingdom with most sequenced species. The sequenced genomes cover fungal species broadly and include industrially, medically and agriculturally important species with very diverse genomes. For these reasons fungal genomes form one of the most attractive eukaryotic datasets for comparative genomics research and method development. Moreover, the possibilities for *in silico *exploration of the diversity of biological functions in these organisms have been revolutionized.

The fungal kingdom is divided in Chytridiomycota, Zygomycota, Glomeromycota, Ascomycota, Basidiomycota and Deuteromycota. Most sequenced fungi belong to Ascomycota, which is again divided in Pezizomycotina, Saccharomycotina and Taphrinomycotina. Pezizomycotina, commonly referred to as filamentous fungi, grow mostly in a filamentous form and degrade biomass to free sugars to be used as source of carbon and energy. In contrast, Saccharomycotina, commonly referred to as yeasts, grow mostly in a unicellular form. They have generally no capability to degrade biomass and accordingly live on free sugars.

Comparative genomics has been applied extensively within the phylum Saccharomycotina (for review see [1]), where a whole genome duplication followed by massive gene loss and specialization has been shown to have occurred [2]. This has been further confirmed by a broader analysis of differential genome evolution in Saccharomycotina species [3] that also demonstrated the usefulness of multi-species protein clustering.

Studies of Pezizomycotina have concentrated on descriptions of individual genomes or comparisons inside a class such as the Eurotiales genomes of *Aspergillus fumigatus *[4], *oryzae *[5] and *nidulans *[6]. *A*. *fumigatus *and *oryzae *had been previously considered as asexual organisms, but comparative analysis showed that their genomes have the same potential for sexual reproduction as *A. nidulans*. Sequencing of the *Neurospora crassa *genome [7] revealed the full effect of the mechanism called Repeat Induced Point mutations (RIP). RIP specifically mutates duplications that are longer than 400 bp and more identical than 80%, thus effectively preventing gene duplications and keeping the genome extremely non-redundant (for review see [8]).

Extensive comparative genomic databases for some groups of eukaryotic species are available such as the Ensembl for mammals [9]. Several comparative databases exist also for fungi such as MIPS [10], CBS Genome Atlases [11], e-Fungi [12], but none offers a level of detail comparable to Ensembl. However, high quality software to set up such a database is freely available from the Generic Genome Browser (GBrowse) [13] and Ensembl projects. Most importantly, these support interfaces to BioPerl [14], which offers currently the most extensive sequence analysis programming library.

Our goal is to explain differences in phenotypes of fungi through differences in their genotypes and to deduce evolutionary processes that have shaped fungi. To this end we have used TRIBE-MCL [15] protein clustering and InterProScan [16] protein domain analysis to compare genome open reading frame (ORF) contents of Pezizomycotina and Saccharomycotina species in a large data set of 33 fungal genomes. TRIBE-MCL divides the genomes comprehensively into protein clusters, while InterProScan detects known protein domains. We have also set up a GBrowse based comparative genomics web interface that enables easy browsing of our data. We detected protein clusters specific to Pezizomycotina, other fungi than Saccharomycotina and Saccharomycotina, and characterised them with Funcat gene classifications [17-19] and Protfun protein function predictions [20].

We found that in Pezizomycotina protein clusters related to plant biomass degradation and secondary metabolism are the only ones with multiple proteins in each species. Pezizomycotina have also an interesting expansion of a transcription factor protein family and a wealth of enzymes and subsequent metabolic capabilities not found in Saccharomycotina. In contrast, Saccharomycotina specific protein clusters are enriched in transcription and mitochondrion related functions.

## Results

### Overview of clustering results

To compare the ORF content of Pezizomycotina and Saccharomycotina we selected a set of 33 species shown in Table 1. 14 of them are Pezizomycotina and 13 Saccharomycotina. In addition one Archeascomycota, four Basidiomycota and one Zygomycota were included as reference species outside the two phyla.

**Table 1:** Genome data

Phylum	Subphylum	Name	Abbreviation	Count of ORFs	Source	WWW	Funcat	InterPro	Protfun
Ascomycota	Archeascomycota	*Schizosaccharomyces pombe*	Spom	4984	Sanger Institute, UK		YES		
Ascomycota	Pezizomycotina	*Aspergillus fumigatus*	Afum	9926	Tigr, USA			YES	
Ascomycota	Pezizomycotina	*Aspergillus nidulans*	Anid	9541	Broad Institute, USA			YES	YES
Ascomycota	Pezizomycotina	*Aspergillus niger*	Anig	11200	Joint Genome Institute, USA				
Ascomycota	Pezizomycotina	*Aspergillus oryzae*	Aory	12062	NITE, Japan			YES	
Ascomycota	Pezizomycotina	*Botrytis cinerea*	Bcin	16446	Broad Institute, USA				
Ascomycota	Pezizomycotina	*Chaetomium globosum*	Cglo	11124	Broad Institute, USA			YES	
Ascomycota	Pezizomycotina	*Coccidioides immitis*	Cimm	10457	Broad Institute, USA				
Ascomycota	Pezizomycotina	*Fusarium graminearum*	Fgra	11640	Broad Institute, USA		YES	YES	YES
Ascomycota	Pezizomycotina	*Magnaporthe grisea*	Mgri	11109	Broad Institute, USA			YES	YES
Ascomycota	Pezizomycotina	*Nectria haematococca*	Nhae	16237	Joint Genome Institute, USA				
Ascomycota	Pezizomycotina	*Neurospora crassa*	Ncra	10082	Broad Institute, USA			YES	YES
Ascomycota	Pezizomycotina	*Sclerotinia sclerotiorum*	Sscl	14522	Broad Institute, USA				
Ascomycota	Pezizomycotina	*Stagonospora nodorum*	Snod	16597	Broad Institute, USA				
Ascomycota	Pezizomycotina	*Trichoderma reesei*	Tree	9997	Joint Genome Institute, USA			YES	YES
Ascomycota	Saccharomycotina	*Ashbya gossypii*	Agos	4726	Ashbya Genome Database, Switzerland			YES	
Ascomycota	Saccharomycotina	*Candida albicans*	Calb	6165	Stanford University, UK		YES	YES	
Ascomycota	Saccharomycotina	*Candida glabrata*	Cgla	5181	Genolevures Consortium, France			YES	
Ascomycota	Saccharomycotina	*Candida guilliermondii*	Cgui	5920	Broad Institute, USA				
Ascomycota	Saccharomycotina	*Candida lusitaniae*	Clus	5941	Broad Institute, USA				
Ascomycota	Saccharomycotina	*Debaryomyces hansenii*	Dhan	6318	Genolevures Consortium, France			YES	
Ascomycota	Saccharomycotina	*Kluyveromyces lactis*	Klac	5327	Genolevures Consortium, France			YES	
Ascomycota	Saccharomycotina	*Pichia pastoris*	Ppas	5999	Integrated genomics, USA				
Ascomycota	Saccharomycotina	*Pichia stipitis*	Psti	5839	Joint Genome Institute, USA				
Ascomycota	Saccharomycotina	*Saccharomyces castellii*	Scas	4677	Saccharomyces Genome Database, USA				
Ascomycota	Saccharomycotina	*Saccharomyces cerevisiae*	Scer	5888	Saccharomyces Genome Database, USA		YES	YES	YES
Ascomycota	Saccharomycotina	*Saccharomyces kluyveri*	Sklu	2968	Saccharomyces Genome Database, USA				
Ascomycota	Saccharomycotina	*Yarrowia lipolytica*	Ylip	6521	Genolevures Consortium, France			YES	YES
Basidiomycota	Hymenomycetes	*Coprinus cinereus*	Ccin	13544	Broad Institute, USA				
Basidiomycota	Hymenomycetes	*Cryptococcus neoformans*	Cneo	7302	Broad Institute, USA				
Basidiomycota	Hymenomycetes	*Phanerochaete chrysosporium*	Pchr	10048	Joint Genome Institute, USA			YES	YES
Basidiomycota	Ustilaginomycetes	*Ustilago maydis*	Umay	6522	Broad Institute, USA		YES		
Zygomycota	Zygomycetes	*Rhizopus oryzae*	Rory	17467	Broad Institute, USA				

Phylum, subphylum, abbreviation used in figures, count of ORFs in the genome, data source and internet address and information, whether Funcat annotation existed and whether InterPro or Protfun analysis was carried out, shown for each species.

**Figure 1:**
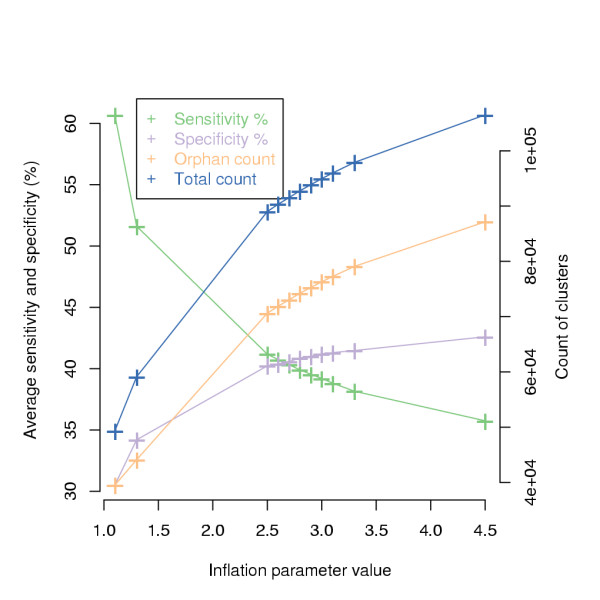
**Selection of the protein clustering parameter "inflation value" (*r*)**. Average sensitivity and specificity percentages (left y-axis) and total count and count of orphan clusters i.e. clusters with only a single member ORF (right y-axis) for clusterings made with different inflation values (x-axis).

To compare the ORF content systematically, we first divided all the ORFs in groups related by sequence similarity. For this we used a protein clustering software TRIBE-MCL [15]. It uses a graph clustering algorithm to cluster proteins based on the results of an all-against-all BLASTP [21]. The results have been shown to correlate well with manual family classifications [15]. The sizes of TRIBE-MCL clusters are influenced by the parameter inflation value (*r*), which is limited to values between 1.1 and 4.5. Subsequently, it effects also the sensitivity, specificity, total count of clusters and count of orphan clusters (i.e. clusters with only one member), which are shown in Figure 1 for different inflation values. We defined sensitivity as the average percentage of how many of the proteins with same InterPro domain structure (see below) are contained in a single cluster. In parallel, specificity was defined as the average percentage of how many of the proteins in a cluster have the same InterPro domain structure [22]. We selected for further analysis a clustering where *r *= 3.1, as a good compromise between sensitivity and specificity.

We used the program InterProScan [23] to detect if proteins from a sub set of genomes (Table 1) had a sequence feature corresponding to an InterPro entry. InterPro is a database of known protein sequence features such as domains, families and active sites, i.e. InterPro entries. In contrast to TRIBE-MCL clustering, InterProScan is not comprehensive as it is limited to known entries accepted to the InterPro database and not uniform because an entry might represent a completely different evolutionary or functional level such as superfamily, subfamily or protein sequence feature such as domain or active site. Thus, TRIBE-MCL clustering allows us to compare genomes in a comprehensive and uniform way, while InterProScan analysis allows a comparison of distributions of specific known sequence features.

**Figure 2:**
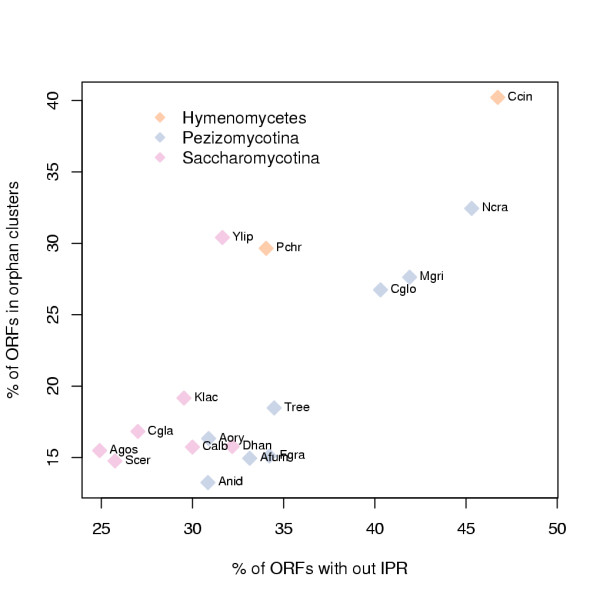
**Unrecognised by InterPro or clustering**. Percentage of ORFs in orphan clusters (y-axis) versus the percentage of ORFs unrecognised by InterPro (x-axis) for species which were analysed with InterProScan. Species are coloured by phyla. Names of the species, plotted as abbreviations beside the data points, are explained in Table 1.

In order to elucidate how well our analysis covers the genomes studied we counted, for the species analysed with InterProScan, the percentage of ORFs in orphan clusters and the percentage of ORFs without any InterPro entries (Figure 2). The Ascomycota genomes with particularly high percentage of orphan clusters in relation to their genome size are *N. crassa *and *Yarrowia lipolytica*. *N. crassa *has also a particularly high percentage of InterProScan unrecognised ORFs. In contrast, the data set includes several closely related members of the Eurotiales; *A. nidulans*, *A. fumigatus*, *A. oryzae *and *A. niger*, which have a particularly low percentage of orphan clusters and InterProScan unrecognised ORFs.

**Figure 3:**
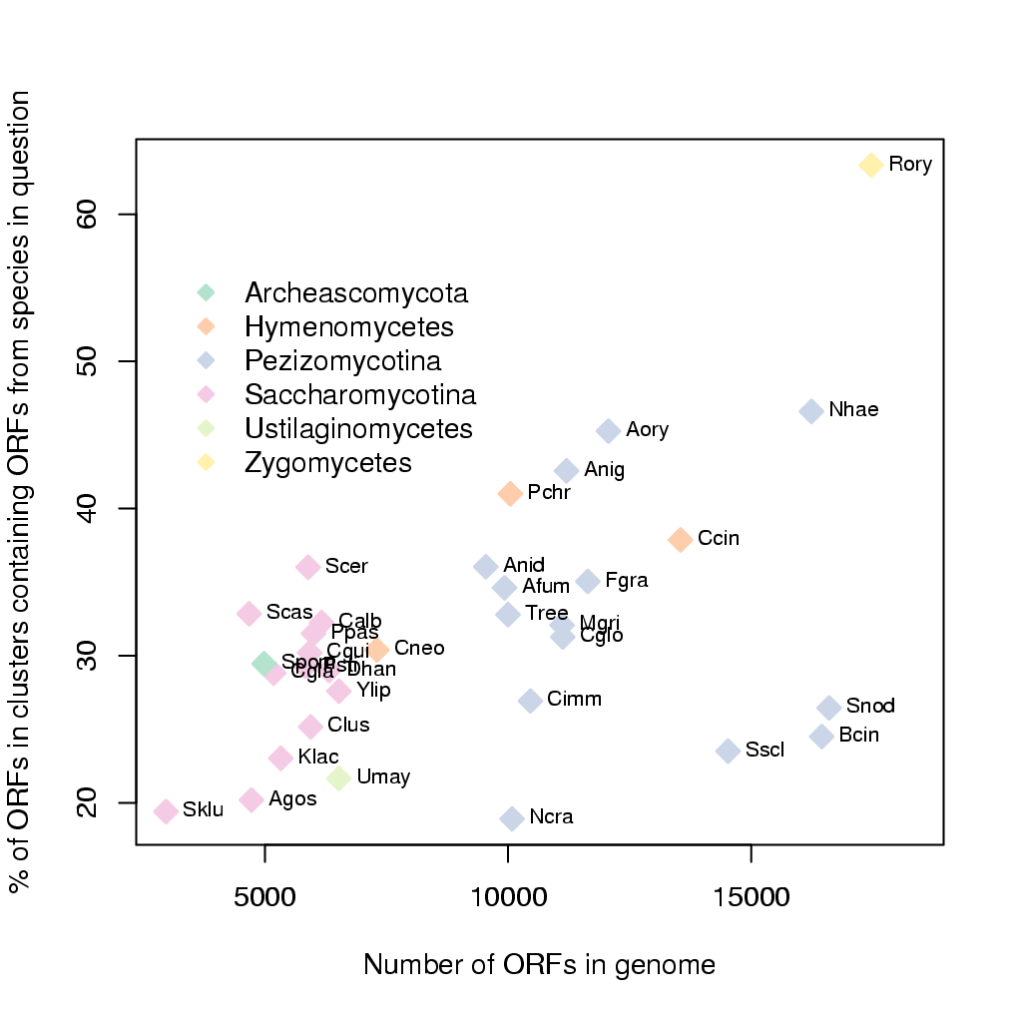
**Genomic ORF redundancy**. Percentage of ORFs in clusters containing more than one ORF from the species in question i.e. genomic ORF redundancy (y-axis) versus the size of the genome in ORFs (x-axis) for each species. See Figure 2 for further details.

To find out how redundant the ORF contents of the genomes studied are, we counted genomic ORF redundancy in each genome (Figure 3). This was defined as the percentage of ORFs in a genome found in clusters with more than one ORF from the same species [3]. Particularly high values of redundancy in relation to their genome sizes are found in the Saccharomycotina species that have undergone a recent genome duplication [2,3] (*S. cerevisiae *and *S. castellii*), while *Candida glabrata*, which also belongs to the genome duplication lineage has an average value. *N. crassa *genome has been shown to have an extremely low level of paralogy due to the RIP mechanism [7], and in accordance it has a particularly low value of redundancy in relation to its genome size. Due to the incomplete state of *S. kluyveri *genome sequence [24] its redundancy is likely to be underestimated.

### Characterization of clusters

**Figure 4:**
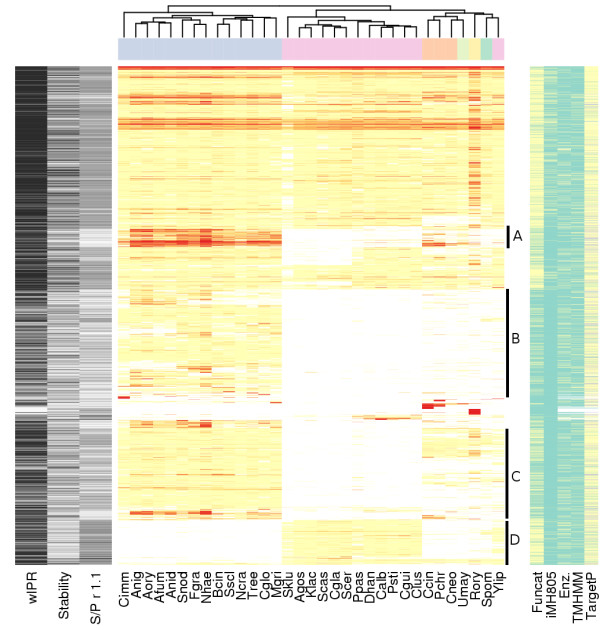
**Protein clustering overview**. See Figure 5 for legend.

**Figure 5:**
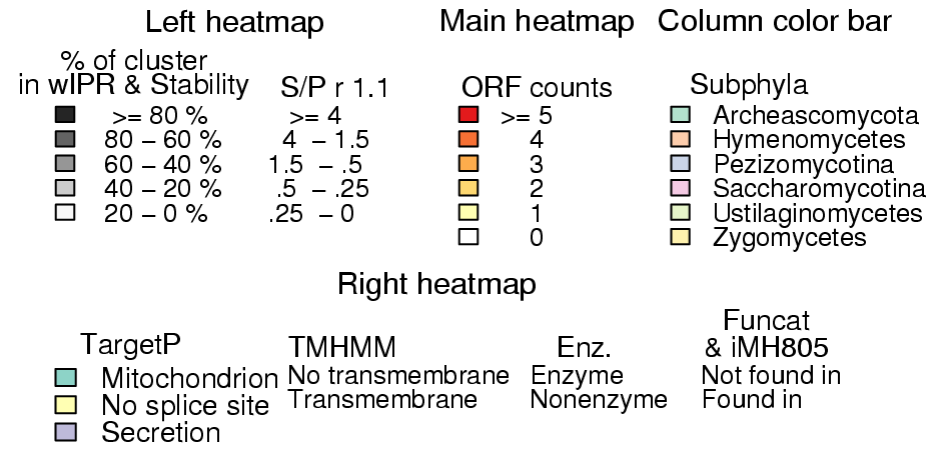
**Legend for figure 4**. A heatmap of clusters with at least ten ORFs. In the main heatmap colour intensity of a cell shows the number of ORFs shown by clusters (rows) and by species (columns). Both rows and columns are ordered by hierarchical clustering to group similar rows or columns together. The dendrogram from hierarchical clustering is shown for columns and the phylum of species is indicated by a column colour bar between the heatmap and the dendrogram. Under the heatmap each species is specified by an abbreviation explained in Table 1. On the left side of the main heatmap a black and white side heatmap shows the percentage of ORFs in a cluster that have an InterPro entry of all cluster's ORFs analysed with InterProScan ("wIPR"), cluster's stability and cluster's Saccharomycotina to Pezizomycotina ratio in a clustering where inflation value (*r*) was 1.1 ("S/P r 1.1"). Stability reflects the ratio of cluster size between a clustering where *r *= 3.1 to that where *r *= 1.1. As Figure 1 shows, when *r *is set to its minimum value (1.1), TRIBE-MCL clustering produces a minimum amount of clusters and orphan clusters. In consequence the clusters are on average larger when *r *= 1.1. The ratio between cluster size *r *= 3.1 and *r *= 1.1 is shown as a percentage. "S/P *r *1.1" reflects the ratio of count of Saccharomycotina ORFs to Pezizomycotina ORFs in a cluster when *r *= 1.1. By comparing "S/P *r *1.1" to the species distribution of a cluster shown on the main heatmap one can see if a cluster retains the Saccharomycotina to Pezizomycotina ratio when *r *= 1.1. On the right side of the main heatmap, a side heatmap shows various functional classifications for the clusters. Whether or not the cluster has a Funcat classification ("Funcat") or has an ORF found in *S. cerevisiae *metabolic model iMH805 is shown ("iMH805"). Whether the proteins in the cluster are predicted by Protfun to have a signal sequence directing them into either mitochondrion or secretion pathway ("TargetP"), have transmembrane domains ("TMHMM") or are predicted to be enzymes is shown ("Enz."). Clusters belonging to regions A: "Pezizomycotina abundant", B: "Pezizomycotina specific", C: "Saccharomycotina absent" and D: "Saccharomycotina unique" are specified by a vertical bar between main and right heatmap.

In order to divide the clusters obtained in our analysis to phylogenetically interesting groups, clusters with at least 10 member ORFs, were ordered by hierarchical clustering based on how many proteins of each species were included in each cluster (Figure 4 and 5 or an excel version [see additional file 1]). We selected four regions with interesting ORF contents for further analysis, A: "Pezizomycotina abundant", B: "Pezizomycotina specific", C: "Saccharomycotina absent" and D: "Saccharomycotina unique". To characterise the content of the regions, they were analysed for enrichment of Funcat categories, Protfun categories and InterPro entries (Tables 2, 3, 4 and 5). Funcat is a hierarchical protein function classification system based on extensive manual annotation [17]. Protfun integrates results from several other protein feature prediction programmes to predict characteristics and functions of a protein based on its sequence [20]. However, Protfun does not use any sequence similarity information, which is essential in attempting to characterise novel protein families unique to fungi. Each cluster's member ORFs were checked for Funcat, InterPro and Protfun assignments and when at least 75% (ProtFun and InterPro) or all (Funcat) ORFs of those for which data was available had the same assignment, it was given to the cluster. If assignments were more heterogeneous, the cluster was assigned as uncategorised for the particular type of assignment. In addition, we checked which clusters contained an *S. cerevisiae *ORF found in the metabolic model iMH805 [25]. The model includes mostly enzyme genes involved in the known primary metabolism of *S. cerevisiae *and also many of their transcriptional regulators. Of the 805 ORFs in iMH805 [25], 737 were successfully mapped to 498 clusters shown in Figure 4.

**Table 2:** Enriched cluster categories in region A: "Pezizomycotina abundant"

Software or database	Database identifier	Category	Count of clusters in region	% of clusters in region	% of all clusters with category	P-value	Author assignment	Figure 13
ProtFun	-	Enzyme/nonenzyme: Enzyme	180	91.0	5	1.60E-11	-	-
ProtFun	-	Enzyme class: Uncategorised	147	74.0	6	1.60E-17	-	-
ProtFun	-	Cellular role: Uncategorised	143	72.0	4	2.10E-02	-	-
Funcat	01	Metabolism	81	41.0	6	8.00E-06	Metabolism	-
ProtFun	-	TargetP: Uncategorised	71	36.0	5	1.40E-04	-	-
Funcat	01.05	C-compound and carbohydrate metabolism	41	21.0	10	1.30E-07	Plant biomass degradation	-
ProtFun	-	TargetP: Secretion	39	20.0	9	1.30E-07	-	-
ProtFun	-	Cellular role: Cell envelope	30	15.0	11	1.20E-08	-	-
Funcat	01.05.01	C-compound and carbohydrate utilization	29	15.0	10	1.30E-05	Plant biomass degradation	-
Funcat	32	Cell rescue, defence and virulence	24	12.0	7	6.50E-03	Virulence/Defence	-
Funcat	01.05.01.01	C-compound, carbohydrate catabolism	22	11.0	22	4.40E-11	Plant biomass degradation	-
ProtFun	-	TMHMM: Uncategorised	20	10.0	7	3.70E-03	-	-
Funcat	01.20	Secondary metabolism	19	10.0	22	1.40E-09	2ary metabolism	-
Funcat	32.05	Disease, virulence and defence	14	7.0	23	1.00E-07	Virulence/Defence	-
Funcat	01.05.01.01.02	Polysaccharide degradation	13	7.0	27	3.50E-08	Plant biomass degradation	-
Funcat	32.05.05	Virulence, disease factors	9	5.0	45	3.20E-08	Virulence/Defence	-
Interpro	IPR001128	Cytochrome P450	8	4.0	42	1.30E-07	2ary metabolism	YES
Interpro	IPR002347	Glucose/ribitol dehydrogenase	8	4.0	21	4.60E-05	2ary metabolism	YES
Funcat	32.07	Detoxification	7	4.0	10	2.20E-02	2ary metabolism	-
Interpro	IPR002198	Short-chain dehydrogenase/reductase SDR	7	3.5	21	1.60E-04	2ary metabolism	YES
Interpro	IPR006094	FAD linked oxidase, N-terminal	5	2.5	56	6.30E-06	2ary metabolism	YES
Interpro	IPR003042	Aromatic-ring hydroxylase	5	2.5	36	8.60E-05	2ary metabolism	YES
Funcat	01.25	Extracellular metabolism	4	2.0	36	7.30E-04	Plant biomass degradation	-
Funcat	02.16	Fermentation	4	2.0	20	8.00E-03	Metabolism	-
Funcat	01.05.01.01.01	Sugar, glucoside, polyol and carboxylate catabolism	4	2.0	19	9.50E-03	Plant biomass degradation	-
Funcat	30.05	Transmembrane signal transduction	4	2.0	12	4.50E-02	Signalling	-
Interpro	IPR011050	Pectin lyase fold/virulence factor	4	2.0	27	1.60E-03	Plant biomass degradation	NO
Interpro	IPR000873	AMP-dependent synthetase and ligase	4	2.0	25	2.10E-03	2ary metabolism	YES
Interpro	IPR008985	Concanavalin A-like lectin/glucanase	4	2.0	24	2.60E-03	Plant biomass degradation	NO
Funcat	32.10.07	Degradation of foreign (exogenous) polysaccharides	3	2.0	50	1.20E-03	Plant biomass degradation	-
Funcat	01.01.09.05	Metabolism of tyrosine	3	2.0	43	2.10E-03	2ary metabolism	-
Funcat	01.05.01.01.09	Aerobic aromate catabolism	3	2.0	43	2.10E-03	Metabolism	-
Funcat	32.10	Degradation of foreign (exogenous) compounds	3	2.0	38	3.30E-03	Plant biomass degradation	-
Funcat	02.25	Oxidation of fatty acids	3	2.0	27	8.80E-03	2ary metabolism	-
Interpro	IPR010730	Heterokaryon incompatibility	3	1.5	75	1.80E-04	Mating	YES
Interpro	IPR003661	Histidine kinase A, N-terminal	3	1.5	43	1.40E-03	Signalling	NO
Interpro	IPR006163	Phosphopantetheine-binding	3	1.5	43	1.40E-03	2ary metabolism	YES
Interpro	IPR005467	Histidine kinase	3	1.5	38	2.20E-03	Signalling	NO
Interpro	IPR001789	Response regulator receiver	3	1.5	33	3.20E-03	Signalling	NO
Funcat	30.05.01.10	Two-component signal transduction system	2	1.0	67	4.90E-03	Signalling	-
Funcat	32.05.05.01	Toxins	2	1.0	50	9.50E-03	2ary metabolism	-
Funcat	01.01.09.05.02	Degradation of tyrosine	2	1.0	40	1.50E-02	2ary metabolism	-
Funcat	01.20.37	Biosynthesis of peptide derived compounds	2	1.0	40	1.50E-02	2ary metabolism	-
Funcat	34.11.11	Rhythm (e.g. circadian, ultradian)	2	1.0	40	1.50E-02	Other	-
Funcat	01.01.09.04	Metabolism of phenylalanine	2	1.0	33	2.30E-02	2ary metabolism	-
Funcat	01.25.01	Extracellular polysaccharide degradation	2	1.0	33	2.30E-02	Plant biomass degradation	-
Funcat	16.05	Polysaccharide binding	2	1.0	33	2.30E-02	Plant biomass degradation	-
Funcat	16.21.05	FAD/FMN binding	2	1.0	33	2.30E-02	2ary metabolism	-
Funcat	01.01.11.03.02	Degradation of valine	2	1.0	29	3.10E-02	2ary metabolism	-
Funcat	30.05.01	Receptor enzyme mediated signalling	2	1.0	25	4.00E-02	Signalling	-
Funcat	01.20.35.01	Biosynthesis of phenylpropanoids	2	1.0	22	5.00E-02	2ary metabolism	-
Interpro	IPR000675	Cutinase	2	1.0	100	1.30E-03	Plant biomass degradation	NO
Interpro	IPR001077	O-methyltransferase, family 2	2	1.0	100	1.30E-03	Regulation	NO
Interpro	IPR002227	Tyrosinase	2	1.0	100	1.30E-03	2ary metabolism	NO
Interpro	IPR008922	Di-copper centre-containing	2	1.0	100	1.30E-03	Dubious	NO
Interpro	IPR012951	Berberine/berberine-like	2	1.0	100	1.30E-03	2ary metabolism	NO
Interpro	IPR000743	Glycoside hydrolase, family 28	2	1.0	67	3.70E-03	Plant biomass degradation	NO
Funcat	01.05.01.01.11	Anaerobic aromate catabolism	1	1.0	100	4.10E-02	Metabolism	-
Funcat	01.20.01.09	Biosynthesis of aminoglycoside antibiotics	1	1.0	100	4.10E-02	2ary metabolism	-
Funcat	01.20.23	Biosynthesis of secondary products derived from L-methionine	1	1.0	100	4.10E-02	2ary metabolism	-
Funcat	01.20.36	Non-ribosomal peptide synthesis	1	1.0	100	4.10E-02	2ary metabolism	-
Funcat	01.20.37.05	Biosynthesis of beta-lactams	1	1.0	100	4.10E-02	2ary metabolism	-
Funcat	02.16.03.03	Heterofermentative pathway and fermentation of other saccharides	1	1.0	100	4.10E-02	Metabolism	-
Funcat	32.05.05.03	Bacteriocins	1	1.0	100	4.10E-02	2ary metabolism	-
Funcat	32.07.09	Detoxification by degradation	1	1.0	100	4.10E-02	2ary metabolism	-
Funcat	34.11.01.01	Light environment response	1	1.0	100	4.10E-02	Signalling	-
Funcat	36.20.18	Plant hormonal regulation	1	1.0	100	4.10E-02	Signalling	-

Software or database that the categories were derived from, category database identifier, category name, count of clusters with the category, percentage of all clusters in this region, percentage of all clusters with the category and significance of enrichment are shown for categories which are enriched in the region (Figure 4) with a p < 0.01. Column "Author assignment" shows an assignment to general themes, based on the respective database, that summarise the InterPro and Funcat categories. While the other assignments are directly based on the databases, "Secondary metabolism" covers entries which are known to participate also in secondary metabolism ([61, 62], Funcat and InterPro). For InterPro entries whether the entry is found from Figure 9 is specified in column "Figure 9". InterPro entries assigned to "Dubious" are entries that InterPro itself considers unreliable.

**Table 3:** Enriched cluster categories in region B: "Pezizomycotina specific"

Software or database	Database identifier	Category	Count of clusters in region	% of clusters in region	% of all clusters with category	P-value	Author assignment	Figure 9
ProtFun	-	TMHMM: No transmembrane	913	81.0	21	5.60E-03	-	-
ProtFun	-	Enzyme/nonenzyme: Nonenzyme	175	16.0	25	1.30E-03	-	-
ProtFun	-	TargetP: Secretion	128	11.0	28	1.90E-05	-	-
ProtFun	-	Cellular role: Cell envelope	93	8.0	35	9.50E-09	-	-
ProtFun	-	Cellular role: Central intermediary metabolism	84	7.0	26	1.20E-02	-	-
Interpro	IPR001138	Fungal transcriptional regulatory protein, N-terminal	41	3.6	51	7.10E-10	Transcription regulation	YES
ProtFun	-	Cellular role: Regulatory functions	37	3.0	39	1.80E-05	-	-
Interpro	IPR007219	Fungal specific transcription factor	25	2.2	37	1.30E-03	Transcription regulation	YES
ProtFun	-	Cellular role: Purines and pyrimidines	27	2.0	28	4.30E-02	-	-
Interpro	IPR001810	Cyclin-like F-box	13	1.2	62	4.00E-05	Macromolecule interaction	YES
Interpro	IPR000637	HMG-I and HMG-Y, DNA-binding	7	0.6	88	9.70E-05	Chromatin	NO
Interpro	IPR001087	Lipolytic enzyme, G-D-S-L	6	0.5	75	1.40E-03	Metabolism	NO
Interpro	IPR006710	Glycoside hydrolase, family 43	6	0.5	75	1.40E-03	Plant biomass degradation	NO
Interpro	IPR005302	MOCO sulphurase C-terminal	3	0.3	100	8.50E-03	Metabolism	NO
Interpro	IPR006209	EGF-like	3	0.3	100	8.50E-03	Extracellular	NO
Funcat	36.20.35	Response to environmental stimuli	3	0.0	75	4.20E-02	Signalling	-

See Table 2 for legend

**Table 4:** Enriched cluster categories in region C: " Saccharomycotina absent"

Software or database	Database identifier	Category	Count of clusters in region	% of clusters in region	% of all clusters with category	P-value	Author assignment	Figure 9
ProtFun	-	TMHMM: No transmembrane	825	82.0	19	8.60E-04	-	-
ProtFun	-	Enzyme/nonenzyme: Enzyme	747	74.0	19	4.50E-02	-	-
ProtFun	-	Cellular role: Uncategorised	722	72.0	20	2.20E-06	-	-
ProtFun	-	TargetP: Mitochondrion	81	8.0	22	4.80E-02	-	-
ProtFun	-	Cellular role: Replication and transcription	9	1.0	38	2.10E-02	-	-
Funcat	01.01.11.04.02	Degradation of leucine	5	1.0	63	1.30E-02	2ary metabolism	-
Funcat	01.01.11.04	metabolism of leucine	5	1.0	50	3.90E-02	2ary metabolism	-
Interpro	IPR001623	Heat shock protein DnaJ, N-terminal	9	0.9	43	7.90E-03	Macromolecule interaction	NO
Interpro	IPR000195	RabGAP/TBC	6	0.6	55	7.30E-03	Secretion	NO
Interpro	IPR000683	Oxidoreductase, N-terminal	6	0.6	55	7.30E-03	Metabolism	NO
Interpro	IPR006091	Acyl-CoA dehydrogenase, central region	4	0.4	100	1.10E-03	Metabolism	NO
Interpro	IPR006092	Acyl-CoA dehydrogenase, N-terminal	4	0.4	100	1.10E-03	Metabolism	NO
Funcat	01.01.11.02.02	Degradation of isoleucine	4	0.0	67	2.00E-02	2ary metabolism	-
Funcat	01.01.11.03.02	Degradation of valine	4	0.0	57	3.90E-02	2ary metabolism	-
Funcat	01.01.03.01.02	Degradation of glutamine	2	0.0	100	4.40E-02	2ary metabolism	-

See Table 2 for legend

**Table 5:** Enriched cluster categories in region C: "Saccharomycotina unique"

Software or database	Database identifier	Category	Count of clusters in region	% of clusters in region	% of all clusters with category	P-value	Author assignment	Figure 9
ProtFun	-	TMHMM: No transmembrane	380	82.0	9	2.90E-02	-	-
ProtFun	-	TargetP: No splice site	306	66.0	10	1.30E-04	-	-
Funcat	11	Transcription	105	23.0	11	2.60E-02	Transcription	-
ProtFun	-	Enzyme/nonenzyme: Nonenzyme	95	20.0	13	7.80E-07	-	-
ProtFun	-	Cellular role: Translation	87	19.0	23	6.20E-20	-	-
Funcat	11.02	RNA synthesis	70	15.0	12	4.70E-02	Transcription	-
ProtFun	-	Enzyme class: Ligase (EC 6.-.-.-)	68	15.0	15	2.40E-06	-	-
ProtFun	-	TargetP: Mitochondrion	54	12.0	14	4.20E-05	-	-
ProtFun	-	Cellular role: Central intermediary metabolism	43	9.0	13	2.00E-03	-	-
ProtFun	-	Cellular role: Amino acid biosynthesis	37	8.0	35	6.80E-15	-	-
Funcat	42.16	Biogenesis of cellular compartment: mitochondrion	34	7.0	21	5.30E-06	Mitochondrion	-
Funcat	12.01.01	Ribosomal proteins	27	6.0	14	3.80E-02	Protein translation	-
ProtFun	-	Cellular role: Energy metabolism	20	4.0	17	1.80E-03	-	-
Funcat	70.16	Subcellular localisation: mitochondrion	18	4.0	16	2.40E-02	Mitochondrion	-
Funcat	11.04.01	rRNA processing	17	4.0	15	3.90E-02	Transcription	-
ProtFun	-	Cellular role: Regulatory functions	15	3.0	16	1.10E-02	-	-
Interpro	IPR011046	WD40-like	14	3.0	18	5.70E-03	Dubious	YES
ProtFun	-	Enzyme class: Isomerase (EC 5. -. -.-)	14	3.0	45	5.30E-08	-	-
Funcat	11.02.01	rRNA synthesis	11	2.0	19	2.40E-02	Transcription	-
ProtFun	-	Cellular role: Biosynthesis of cofactors	7	2.0	37	6.00E-04	-	-
ProtFun	-	Cellular role: Replication and transcription	7	2.0	29	2.80E-03	-	-
Funcat	18.02.09	Regulator of transcription factor	5	1.0	31	1.50E-02	Transcription regulation	-
Interpro	IPR006596	Nucleotide binding protein, PINc	3	0.6	60	5.20E-03	Nucleotide binding	NO
Interpro	IPR000992	Stress-induced protein SRP1/TIP1	2	0.4	100	7.10E-03	Stress response	NO
Interpro	IPR007757	MT-A70	2	0.4	100	7.10E-03	Nucleotide binding	NO

See Table 2 for legend

Overall, 57% of the clusters with at least 10 members were assigned with a Funcat category, but the cover of Funcat differs greatly between the regions (Figure 4). The respective percentages for the selected regions are, A: "Pezizomycotina abundant" 61%, B: "Pezizomycotina specific" 20%, C: "Saccharomycotina absent" 43% and D: "Saccharomycotina unique" 81%. Also, over 95% of the clusters having members from all species shown at the upper part of main heatmap of Figure 4 have a Funcat category. The ORFs of the *S. cerevisiae *metabolic model iMH805 [25] are distributed even more strikingly. Most clusters having these ORFs have members from all species. In addition 7% of the clusters in D: "Saccharomycotina unique" have an iMH805 [25] ORF (see below).

198 clusters were assigned to the region A: "Pezizomycotina abundant". They have usually more ORFs in Pezizomycotina than in other phyla and none in Saccharomycotina (Figure 4). The categories of clusters significantly enriched in this region compared to other clusters are shown in Table 2. Clusters of the region contain mainly predicted enzymes. 20% of the clusters were predicted to have a secretion pathway signal sequence and 36% of the clusters had heterogeneous signal sequence predictions and were assigned as "TargetP: Uncategorised" (Table 4). Thus, it is likely that more than 20% of the clusters are actually secreted. The enriched Funcat categories of the clusters are mainly related to plant biomass degradation and secondary metabolism (Table 2). Enriched InterPro entries of the clusters are also mainly in categories connected to secondary metabolism and plant biomass degradation. However, proteins with domains related to secondary metabolism might also have other functions. For example, the methylation carried out by "IPR01077: O-methyltransferases family 2" domains can function in transcription regulation or aflatoxin biosynthesis [26].

The 1130 clusters of the region B: "Pezizomycotina specific" have usually just one ORF in each Pezizomycotina species and none in the other phyla (Figure 4). Most proteins in these clusters were predicted not to have transmembrane regions and 16% of them were predicted not to be enzymes. 11% of the clusters were predicted to have a secretion pathway signal sequence. The cellular role predictions "Cell envelope", "Regulatory functions", "Central intermediary metabolism" "Purines and pyrimidines" show significant enrichment. The Funcat classification category "response to environmental stimuli" is the only one significantly enriched (Table 3). Enriched InterPro entries IPR001138 and IPR007219 (Table 5), are transcription factor domains that commonly occur together (see chapter "PCA of InterPro entry counts" below). Otherwise the enriched entries are functionally diverse.

**Figure 6:**
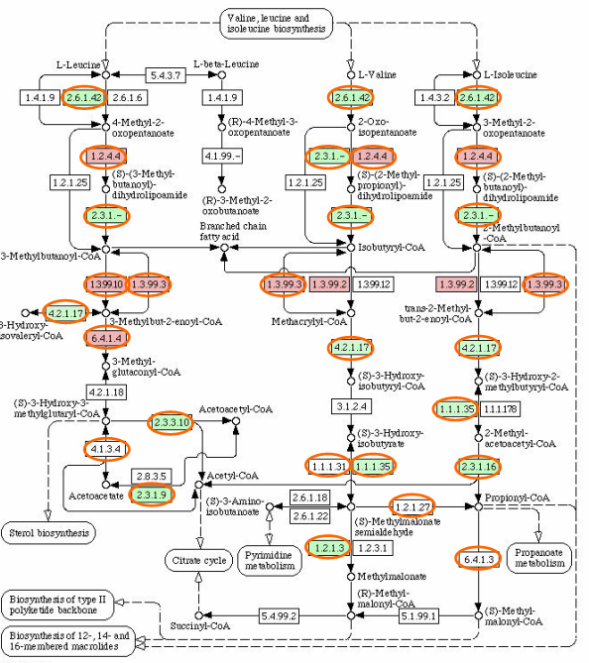
**The KEGG metabolic pathway "Valine, leucine and isoleucine degradation"**. Enzymes found in *S. cerevisiae *according to SGD and KEGG are filled with **green **and in *M. grisea *according to KEGG circled in **orange**. Enzymes found in clusters corresponding to Funcat categories 01.01.11.02 -01.01.11.04 enriched in the region C: "Saccharomycotina absent" in Table 3 are filled with **pink**.

The 1010 clusters of the region C: "Saccharomycotina absent" have usually just one ORF in each Pezizomycotina species and none in Saccharomycotina (Figure 4). They were predicted mainly not to have transmembrane regions and 74% were predicted to be enzymes. No cellular role could be predicted for 72% of the clusters (Table 4). Several Funcat amino acid degradation categories are enriched in these clusters (Table 4). However, all these cases correspond to only five clusters that have different overlapping categories. The Enzyme Commission (EC) numbers found in Funcat for the proteins in these clusters all map to the KEGG [27] pathway "Valine, leucine and isoleucine degradation" (Figure 6). According to SGD and KEGG *S. cerevisiae *has only few of the enzymes in this pathway, while *M. griseae *has most of them. InterPro entries enriched in the clusters of the region are mainly related to metabolism (Table 4). The two Acyl-CoA dehydrogenase entries are in the same clusters and correspond to proteins with EC: 1.3.99.3. shown in Figure 6.

The 466 clusters of the region D: "Saccharomycotina unique" have usually just one ORF in each Saccharomycotina species and none in the other phyla (Figure 4). These clusters were mainly predicted not to have transmembrane regions, and 66% were predicted not to have any signal sequence. 20% of the clusters were predicted not to contain enzymes, while 15% were predicted to be ligases. Several predicted cellular roles were also enriched (Table 4). Two of the four InterPro entries enriched in the clusters of this region are related to nucleotide binding (Table 5).

The Funcat categories enriched are either related to mitochondrion, transcription or translation (Table 3). Categories "Biogenesis of cellular components: mitochondrion" and "Ribosomal proteins" overlap. Of the 34 clusters in "Biogenesis of cellular components: mitochondrion", 28 contain an *S. cerevisiae *ORF described by *Saccharomyces *Genome Database (SGD) [28] as mitochondrial ribosomal component of the small or large subunit. Of these ORFs, 26 are found in clusters with the category "Ribosomal proteins".

In contrast, five clusters with the category "Subcellular localisation: mitochondrion" contain *S. cerevisiae *proteins not related to ribosomes that have been localised to mitochondrion in a genome wide analysis [29]. For the other clusters with "Subcellular localisation: mitochondrion" Funcat proposes, based on sequence similarity, that they could be localised to mitochondrion. The function of the ORFs found in the clusters with this enriched category is not known. However, YJL082w (IML2) found in the clusters has been shown to have a physical interaction with YLR014c (PPR1) [30], a positive transcriptional regulator of the pyrimidine biosynthesis pathway [31]. Also, according to InterProScan YHF198c (FMP22) found in the clusters is a member of the "Purine/pyrimidine phosphoribosyl transferase" family (IPR002375), like the YML106W (URA5) and YMR271C (URA10) genes involved in the actual pyrimidine biosynthesis pathway. This evidence suggests that Saccharomycotina could have a novel mitochondrial pyrimidine related pathway not found in Pezizomycotina.

In addition to enriched cluster categories, the region D: "Saccharomycotina unique" is characterised by 34 clusters, which have a gene found in the *S. cerevisiae *metabolic model iMH805 [25]. Interestingly, instead of actual metabolic enzymes, 13 of these are transcription factors. Besides "01 Metabolism", the Funcat category most enriched among these clusters in comparison to all other clusters is "01.07.01 biosynthesis of vitamins, cofactors and prosthetic groups" (p < 2,2*10^e-6^, 7 clusters).

### PCA of InterPro entry counts

The analysis of the TRIBE-MCL clusters presented above dealt only with clusters of at least ten member ORFs. However, the clustering produced 77.278 clusters with only a single member and 11.727 of these had InterPro entries (Figure 2). In order to find differences in counts of ORFs with InterPro entries or InterPro entry structures between the species, independently of TRIBE-MCL clusters, we carried out a Principal Component Analysis (PCA). InterPro entry structures were constructed to each ORF by reducing overlapping and redundant InterPro entries into a single most specific one and concatenating these in the order they appear in the protein sequence. This enabled us to differentiate between InterPro entries appearing alone in an ORF from those that commonly appear together in an ORF.

PCA finds principal components (PC), i.e. major sources of variation between the species and positions of the original samples and the loadings of individual InterPro entries or InterPro entry structures on the PCs. The PCs are named so that the PC explaining the highest amount of variation is called PC1, the PC explaining the next highest amount of variation PC2 etc. A loading represents the contribution of an InterPro entry or an InterPro entry structure in forming a PC. For example, an InterPro entry with the lowest loading on PC1 is the largest contributor to positions low on PC1.

**Figure 7:**
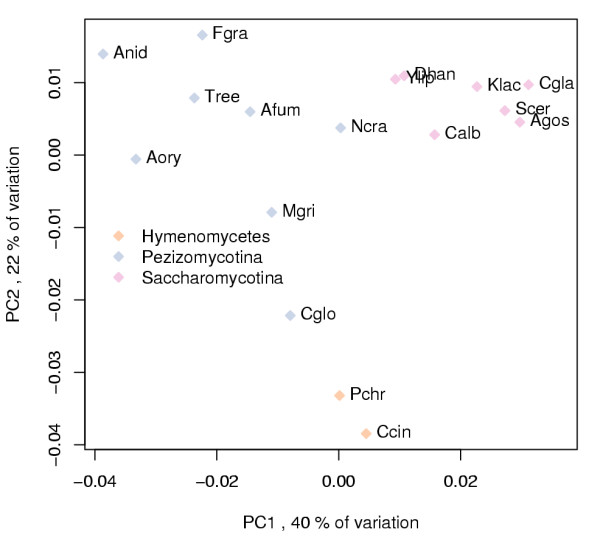
**PCA of counts of ORFs with an InterPro entry**. Positions of species analysed with InterProScan on the two PCs that explain the largest amount of variation in the counts of ORFs with an InterPro entry. Species abbreviations are explained in Table 1 and data points are coloured by phyla.

We found that in a PCA of counts of ORFs with InterPro entries, PC1 explains 40% of all the variation in the data while PC2 explains 22%. The positions of the species on these two PCs are shown in Figure 7. Pezizomycotina are separated from Saccharomycotina on PC1. Between these phyla *N. crassa *and *Y. lipolytica *have the least difference and Hymenomycetes are similarly in the middle of PC1. This means that in counts of ORFs with InterPro entries the difference between Pezizomycotinaand Saccharomycotina is the largest source of variation and InterPro entries with lowest loadings on PC1 are the most abundant in Pezizomycotina in comparison to Saccharomycotina and vice-versa for highest loadings. Hymenomycetes are separated from other fungi on PC2. An almost identical result was achieved by using counts of ORFs with an InterPro entry structure instead (data not shown).

**Figure 8:**
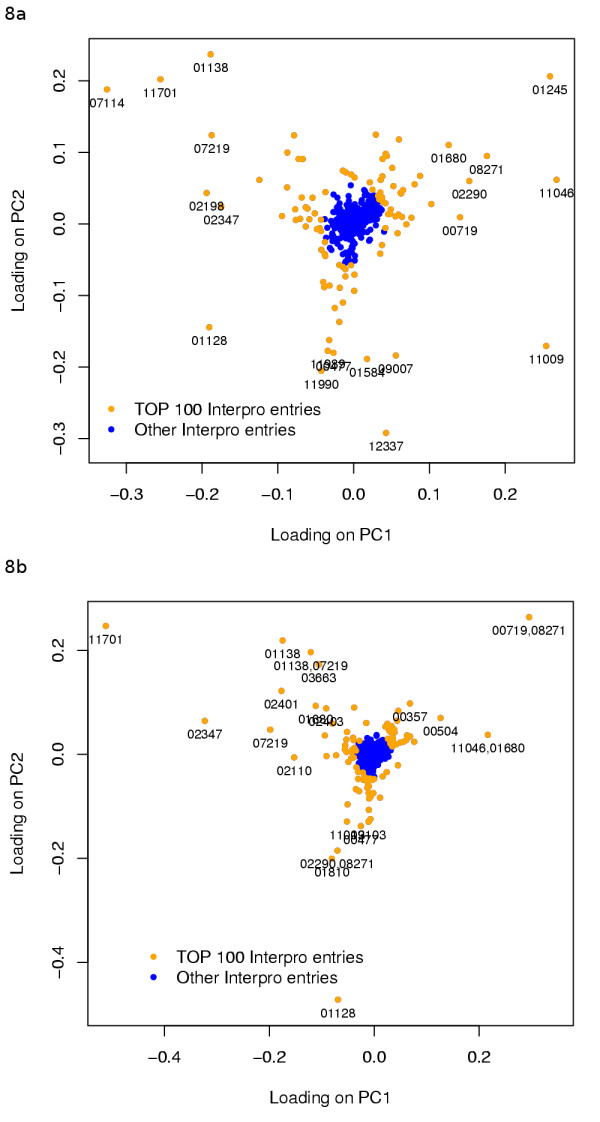
**PCA loadings of InterPro entries and InterPro entry structures**. PCA loadings of InterPro entries (a) or InterPro entry structures (b) of the two PCs that explain the largest amount of variation in the counts of ORFs with an InterPro entry. The PCA for InterPro entries (a) is shown in picture 11, for InterPro entry structures (b) data not shown. 100 InterPro entries or InterPro entry structures having the most extreme PCA loadings on the two PCs shown are coloured with orange (TOP 100), while the rest are 4373 InterPro entries and 16319 InterPro structures are in blue. InterPro entries identifiers are shown for 20 most extreme PCA loadings.

**Figure 9:**
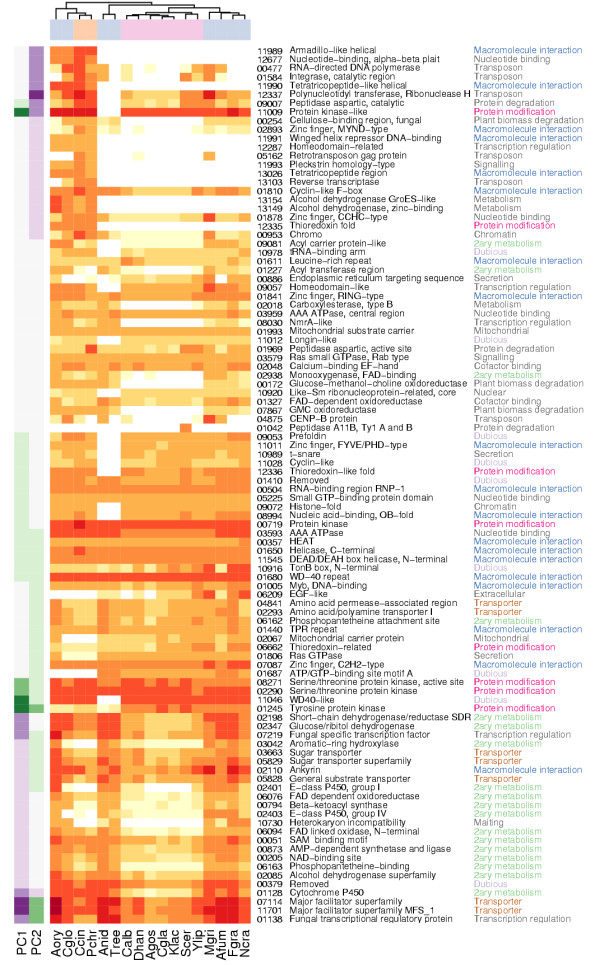
**TOP 100 InterPro entries**. See Figure 10 for legend.

**Figure 10:**
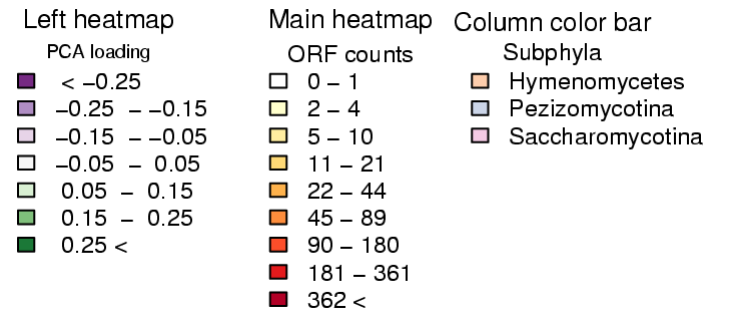
**Legend for figure 9**. A heatmap of counts of ORFs with an InterPro entry for the TOP 100 entries from Figure 8a. In the main heatmap colour intensity of a cell shows the number of ORFs with an InterPro entry shown by entries (rows) and by species (columns). Both rows and columns are ordered by hierarchical clustering to group similar rows or columns together. Columns were clustered with counts of ORFs while rows were clustered with the entry PCA loadings (Left side heatmap and Figure 8a). The dendrogram from hierarchical clustering is shown for columns and the phylum of species is indicated by a column colour bar between the heatmap and the dendrogram. Under the heatmap each species is specified by an abbreviation explained in Table 1. Left side heatmap shows the loading of the entry as in Figure 8a. Interpro entry identifier ("IPR id.", "IPR0" removed from beginning), name ("IPR name") and "Author assignment" are shown for each entry. The "Author assignment" is an assignment to general themes that summarise the individual categories based on the InterPro database. While the other assignments are directly based on the InterPro, "Secondary metabolism" covers entries which are known to participate also in secondary metabolism ([61, 62] and InterPro). InterPro entries assigned to "Dubious" are entries that InterPro itself considers unreliable.

To understand which individual InterPro entries (Figure 8a) or InterPro entry structures (Figure 8b) contribute most to the difference between the species we studied InterPro entry and InterPro entry structure loadings on PC1 and PC2 of their respective PCAs. We selected the 100 InterPro entries or InterPro entry structures with most extreme loadings (TOP 100) and visualised the counts of ORFs of the TOP 100 InterPro entries from Figure 8a as a heatmap (Figure 9 and 10, or an excel version [see additional file 2]).

Based on the distributions of the loadings less than hundred InterPro entries or InterPro entry structures actually notably contribute to the positions of the species on PC1 and PC2 (Figure 8a and 8b). The TOP100 InterPro entries are related to a wide variety of biological functions, but the five most common, with their counts, are: "Macromolecule interaction, 19", "Secondary metabolism, 18", "Dubious, 9", "Protein modification, 8" and "Transporter, 7". InterPro entries assigned to "Dubious" are entries that InterPro itself considers unreliable.

The InterPro entry structures contributing most to PC1 and PC2 and having several InterPro entries that appear together are "IPR001138: Fungal transcriptional regulatory protein, N-terminal, IPR007219: Fungal specific transcription factor", "IPR000719: Protein kinase, IPR008271: Serine/threonine protein kinase, active site", "IPR011046: WD40-like, IPR001680: WD40 repeat" and "IPR002290: Serine/threonine protein kinase, IPR008271: Serine/threonine protein kinase, active site" (Figure 8b). These seven InterPro entries belong also to the TOP100 individual entries shown on Figure 8a and 8.

IPR001138 is a DNA binding N-terminal zinc binuclear cluster (Zn_2_Cys_6_) domain and IPR007219 a C-terminal domain commonly found in proteins with IPR001138. Both are found in many fungal transcription factors such as *S. cerevisiae *GAL4 [32], *A. niger *xlnR [33] and *Aspergillus flavus *aflR [34]. Of proteins with IPR001138, 39% have also an IPR007219. On the average a Pezizomycotina has three times more ORFs with IPR001138 than a Saccharomycotina.

### Browsable fungal comparative genomics database

Automatic classifications of translated ORFs to families based on similarity to known families or *de novo *classifications by translated ORF clustering are not perfect. We have used InterProScan [16] to find out if an ORF is a member of a known family or has some known sequence feature like a domain or an active site i.e. has an InterPro entry. We also used TRIBE-MCL [15] to cluster ORFs based purely on sequence similarity. Our database aims to integrate these two classifications in the context of fungal comparative genomics and to allow easy browser access to the data.

**Figure 11:**
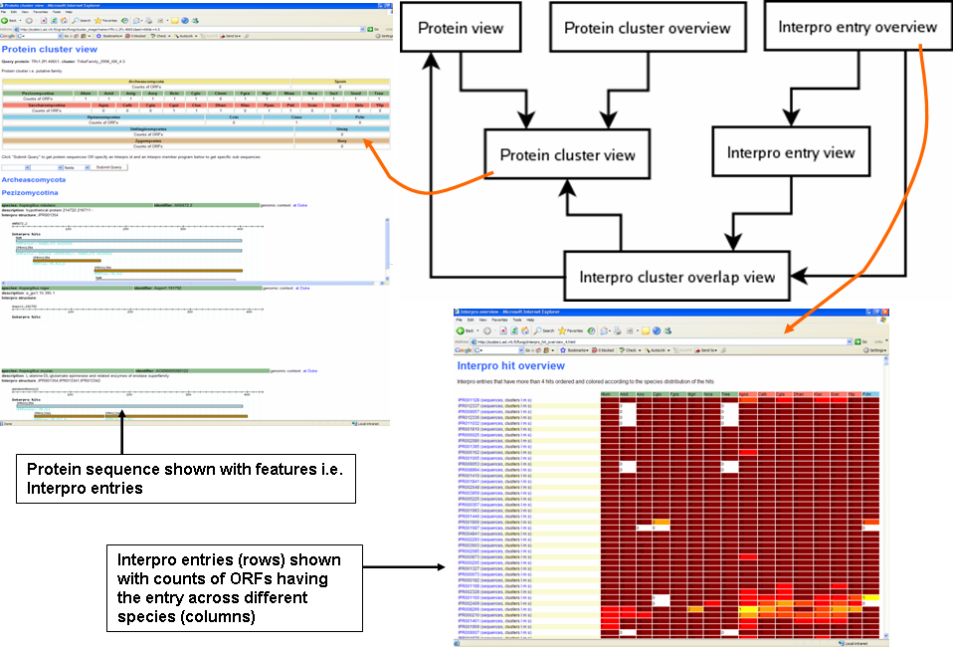
**Schema and screenshots of the browsable fungal comparative genomics database**. The schema on the upper right corner shows links between different browser views to the database. Additionally two example screenshots are shown. See text for further details.

Figure 11 shows a schematic of browser views and the links between them and example screenshots. "Protein view" shows an individual ORF with its InterPro entries. It is mainly used to access "Protein cluster view" that shows all the member ORFs of a cluster with InterPro entries found in them. "InterPro entry view" is identical to "Protein cluster view", except that it shows all ORFs with a given InterPro entry. "Protein cluster overview" and "InterPro entry overview" show a heatmap where clusters or InterPro entries are rows, and the columns show the count of ORFs in each species. The two overviews are used to find clusters or InterPro entries with similar phylogenetic profiles. "InterPro cluster overlap view" shows for a given InterPro entry a table of all clusters whose members have the entry.

The sizes of the TRIBE-MCL [15] clusters are influenced by the parameter inflation value. For an overall comparison of genomes we selected the inflation value 3.1, because it gives a good compromise between clustering sensitivity and specificity (Figure 1). However in individual cases this might not be the best value. Users can specify different inflation values while browsing the database.

## Discussion

### Overview of clustering results

We have analysed the ORF content of Pezizomycotina and Saccharomycotina species with sequence similarity based protein clustering. The main clustering parameter, inflation value (*r*), was selected as a compromise between sensitivity and specificity of clustering. Consequently, the number of ORFs in clusters with only a single member (orphan clusters) is twice as large with the selected (*r *= 3.1) than with the lowest (*r *= 1.1) inflation value (Figure 1). The clusters were then divided in phylum specific groups for further analysis. The column "S/P *r *1.1" in Figure 4 shows that the ratio of Saccharomycotina to Pezizomycotina ORF counts, i.e. the phylum specificity, is largely maintained in the clusters even in *r *= 1.1 clustering. This holds especially well for A: "Pezizomycotina abundant" and D: "Saccharomycotina unique" clusters, while other regions have more variation.

As expected, *N. crassa *due to its extreme RIP mechanism, has the lowest genomic ORF redundancy among Pezizomycotina (Figure 3) [7]. Variable RIP activity could also explain the separation of Sordariomycetes species (*T. reesei*, *F. graminerum, C. globosum*, *M. grisea *and *N. crassa*) in to two groups in regard to counts of unrecognised ORFs (Figure 2). It has been proposed that *Aspergillus *species and *M. grisea *could have a milder RIP like mechanism [35,36]. Our analysis does not exclude this possibility, but it is clear that the *N. crassa *genome is particular among fungi. *N. crassa *and *S. cerevisiae *are probably the most commonly used fungal model organisms and thus it is notable that with regard to genomic ORF redundancy they have the most exceptional genomes within the analysed large fungal genome set.

### Characterisation of cluster regions

From the protein clustering results presented in Figure 4 we selected four interesting regions containing clusters with particular phylogenetic distributions. The clusters in these regions were then searched for enrichment of Protfun and Funcat category and InterPro entry annotations.

Clusters in the region A: "Pezizomycotina abundant" have usually more ORFs in Pezizomycotina than in other phyla and none in Saccharomycotina(Figure 6). The results of all three annotation analyses of these clusters agree well (Table 2). This region contains relatively well characterised enzymes involved either in secondary metabolism or in extracellular plant biomass degradation machinery, but Funcat analysis finds far more clusters involved in plant biomass degradation than InterPro analysis. However, InterPro provides much less detail on glycosyl hydrolases involved in lignocellulose degradation, than for example the specialised Carbohydrate-active enzymes (CAZY) database [37].

Many of the enzymes in clusters of this region, such as cytochrome P450s, glycoside hydrolases and tyrosinases are at the edge of the metabolic network involved in synthesis of the final secreted metabolites or degradation of the external sources of carbon. Consequently, the high sequence homology and the resulting larger clusters of A: "Pezizomycotina abundant" proteins could be due to recent protein family expansions to give more possibilities for the fungi to interact with the environment.

Clusters in the region B: "Pezizomycotina specific" have usually just one ORF in each Pezizomycotina species and none in the other phyla (Figure 4). In comparison to A: "Pezizomycotina abundant" clusters, far fewer member ORFs have any InterPro entries (Figure 4, column "wIPR") and very few InterPro or Funcat annotations show enrichment (Tables 3). The significant enrichment of the Protfun "Cellular role: Regulatory functions" and InterPro entries "IPR001138: Fungal transcriptional regulatory protein, N-terminal" and "IPR007219: Fungal specific transcription factor" coincide well.

Clusters in the region C: "Saccharomycotina absent" have usually just one ORF in each Pezizomycotina species and none in Saccharomycotina (Figure 4). According to Protfun predictions these are mostly enzymes. In comparison to B: "Pezizomycotina specific" clusters, more member ORFs have some InterPro entries (Figure 4, column "wIPR"), but still neither InterPro entry nor Funcat category enrichment provide much detail, except for proteins found in the KEGG pathway "Valine, leucine and isoleucine degradation" (Figure 6). The "EC: 1.3.99.3" acyl-CoA dehydrogenase, detected by InterPro and Funcat enrichment analysis, is a key enzyme for fatty acid β-oxidation in mitochondria. The capability for fatty acid β-oxidation in mitochondria has been shown experimentally for *A. nidulans *[38] and proposed by genome analysis for several other Pezizomycotina species [6,38]. However, Saccharomycotina can apparently only carry out fatty acid β-oxidation in peroxisomes [39-42]. The proteins detected by Funcat enrichment analysis and found in *M. grisea *are according to KEGG linked to polyketide synthesis (Figure 6). A reason for the other fungi than Saccharomycotina to have this capability while the Saccharomycotina can do without, could be the ability to provide precursors from the fatty acid and amino acid metabolism to the polyketide synthesis.

B: "Pezizomycotina specific" and C: "Saccharomycotina absent" clusters contain proteins of low homology that subsequently divide into small equally sized clusters. They seem to contain proteins more connected in cellular metabolism and regulation networks than the clusters in the region A: "Pezizomycotina abundant" and thus be under more pressure not to expand to keep the topology of the networks conserved. This would allow the sequences to diverge in time without simultaneous expansion of the families and result into small clusters broadly conserved over Pezizomycotina.

In addition, based on the distribution of the clusters with ORFs found in *S. cerevisiae *metabolic model iMH805 [25], it is clear that over 90% of these ORFs are well conserved in all fungal species. These form the conserved core of the fungal metabolic network. Metabolism related ORFs in B: "Pezizomycotina specific" and C: "Saccharomycotina absent" could then represent a mid layer around the core such as the ORFs shown in Figure 6, that link primary metabolism to secondary metabolism and biomass degradation machinery. However, Protfun predicts that most B: "Pezizomycotina specific" ORFs are not related to metabolism (Table 4), rather they could be involved for example in Pezizomycotina specific morphology. Actual biomass degradation and secondary metabolism machineries, i.e. A: "Pezizomycotina abundant" ORFs, could then form the edges of the network. Being on the outer rim of the metabolic network they could be subjected to minimal evolutionary constraints for conserving network topology and could be directly subjected to various evolutionary pressures by the changing environments and thus evolve fast.

Proteins involved in secondary metabolism, glycoside hydrolases and predicted secreted uncharacterised ORFs are found in for example subtelomeric regions of *Magnaporthe oryzae *[43]. Subtelomeric regions appear to have a potential for faster evolution than many other chromosomal regions [43-45]. Consequently, subtelomeric positioning could provide a mechanism to explain expansion of A: "Pezizomycotina abundant" like protein families.

Clusters in the region D: "Saccharomycotina unique" have usually just one ORF in each Saccharomycotina species and none in the other phyla (Figure 4). Various Protfun cellular role predictions are enriched in these clusters, among them translation, transcription and mitochondrion related, found also in Funcat enrichment analysis. Strikingly, clusters with the Funcat category "42.16: Biogenesis of cellular components: Mitochondrion" correspond to at least 28 different components of mitochondrial ribosomal protein complexes. However, homologous Pezizomycotina ORFs are found in the region "B: Pezizomycotina specific". The protein sequences in different families of mitochondrial ribosomal proteins have diverged so much between Pezizomycotina and Saccharomycotina that TRIBE-MCL splits the families in different clusters. This implies that these complexes have diverged significantly between Pezizomycotina and Saccharomycotina.

### Comparison of clustering and PCA results

In addition to clustering, a PCA of counts of ORFs with an InterPro entry was done. We searched for known protein sequence features (domains, families, and active sites etc., i.e. InterPro entries) with the program InterProScan from a subset of genomes in our data set (Table 1), counted ORFs having an entry and analysed the counts with PCA. The function of the 100 InterPro entries with most differences between the species analysed (TOP 100) was studied in detail.

TRIBE-MCL clustering and InterPro analyses complemented each other well. Proteins that are conserved in several species could be studied comprehensively regardless of how well they have previously been described in InterPro by clustering (Figure 4). However, the TOP 100 InterPro entries contain interesting details not detected by clustering, such as the expansion of major facilitator superfamilies (MFS) and macromolecule interaction related protein families in Pezizomycotina and the distribution of transposon related proteins among Pezizomycotina (Figure 9). MFS transporters lie in clusters with ORFs from all species and due to this their expansion was not detected clearly by the clustering analysis (Figure 4). In contrast, enrichment of "IPR001138: Fungal transcriptional regulatory protein, N-terminal" was detected. However, the full importance of this expansion was only revealed by PCA, because there are 614 clusters with less than 10 members that have an IPR001138. As InterPro domain analysis uses searches optimised for individual protein families or domains, it can detect well both families with high internal sequence similarity such as MFS transporters and with low internal sequence similarity such as IPR001138, if the family has been previously well characterised.

The InterPro entries among the TOP 100 with the lowest values on PC1, thus most abundant in Pezizomycotina in comparison to Saccharomycotina (Figures 8 and 9), overlap with cluster InterPro entry enrichment of A: "Pezizomycotina abundant" and B: "Pezizomycotina specific" clusters (Tables 2 and 3). The major difference between clustering and PCA results is that in clustering transcription factors belong to B: "Pezizomycotina specific", and secondary metabolism related clusters fall into A: "Pezizomycotina abundant", while PCA makes no such distinction. MFS transporters have also very negative values on PC1 (Figure 9). They are transporters found for example in fungal secondary metabolite clusters for gliotoxin [46] or trichothecene [47] synthesis. As secondary metabolism seems to be the major function connecting InterPro entries with most negative values on PC1, it could be expected that "IPR001138: Fungal transcriptional regulatory protein, N-terminal" family has expanded to control secondary metabolism and that MFS transporters would have expanded to export the produced metabolites. However, likely targets are also the many plant biomass degradation related clusters found in A: "Pezizomycotina abundant", for example IPR001138 transcription factor *A. niger *xlnR [33] is known to regulate cellulase and hemicellulase genes and *A. flavus *aflR [34] regulates a secondary metabolism pathway. Furthermore, Pezizomycotina specific features like ascocarp formation or other yet incompletely described processes could involve these transcription factors.

Hierarchical clustering of species' cluster ORF (Figure 4) or InterPro entry (Figure 9) counts produce different species similarity dendrograms (shown on top of the heatmap in both figures). In Figure 4, the tree constructed from clusters with at least ten ORFs, resembles closely the one presented by NCBI Taxonomy database [48]. However, *Y. lipolytica*, expected to be the first species to diverge from the Saccharomycotina lineage (for review see [1]), is separated from other yeasts. This could imply that *Y. lipolytica *ORFs and ORF content of conserved cellular functions resemble more those of other fungi than Saccharomycotina. In the tree constructed from the TOP 100 InterPro entries Saccharomycotina are grouped together while Pezizomycotina are not divided as expected (Figure 9). For example, the Eurotiales are grouped together in Figure 4, although they are separated in Figure 9. This could mean that even though the ORF contents among Eurotiales are very similar at large, in regard to protein families contributing most to the differences between the fungi studied, individual Eurotiales can resemble more other less related species. For example the human pathogen *A. fumigatus *is more similar to the plant pathogen *M. griseae *than to other Eurotiales.

## Conclusion

In conclusion we have carried out a detailed comparison of the ORF content of Pezizomycotina and Saccharomycotina species and found previously well described and novel interesting differences. Our analysis includes fundamentally different but complementary databases and methods. Due to this we are able to support our conclusions with results from independent analysis. The use of a large multi genome dataset further emphasises the credibility of the results.

We highlight promising targets for future experimental studies such as the expanded transcription factor family and potential novel secreted protein families in Pezizomycotina. In general the proteins specific to and well conserved in Pezizomycotina should be studied further as they are largely unknown. We propose an evolutionary explanation for the discovered Pezizomycotina genome features. However, confirmation of this requires detailed comparisons of the chromosomal localisations of the genes in question and of the fungal metabolic and regulatory network.

## Methods

Sequence and cluster analyses and annotation, database construction and interactions and usage of various bioinformatics software was done with custom Perl scripts using when possible the BioPerl [14] libraries. Final data analysis and visualisation of results was done with custom R scripts using the Bioconductor libraries [49] when possible.

### Retrieval and analysis of sequence data

Protein sequences of predicted open reading frames (ORF), in fasta format, were retrieved from various sequencing centres as indicated in Table 1. They have been published in the following articles: *S. pombe *[50], *A. fumigatus *[4], *A. nidulans *[6], *A. oryzae *[5], *M. grisea *[36], *N. crassa *[7], *A. gossypii *[51], *C. albicans *[52], *C. glabrata *[3]*, D. hansenii *[3]*, K. lactis *[3], *S. castellii *[53], *S. cerevisiae *[54], *S. kluyveri *[53], *Y. lipolytica *[3], P.* chrysosporium *[55], *U. maydis *[56], in addition *P. pastoris *was purchased from ERGO [57]. Sequences were inserted to Oracle database (Oracle) according to the BioPerlDB schema. The Additional file 3 maps each sequence identifier to its species enabling the use of other Additional files [see additional file 3].

To find functional and pathway annotations for proteins Funcat [17-19] annotations were downloaded from MIPS [58] for genomes indicated in Table 1.

Proteins from a subset of genomes (Table 1) were analysed with InterProScan [16] to find features from their sequence, i.e. InterPro [59] entries. InterPro entries can have hierarchical parent-child relationship. For example IPR001236 "Lactate/malate dehydrogenase " is a parent of IPR010097 "Malate dehydrogenase, NAD-dependent". For each protein an InterPro entry structure, i.e. a vector of InterPro entry identifiers in the order they appear on the sequence, was constructed. From non-identical entries overlapping along protein sequence only the most specific was selected in case a parent-child relationship existed between them. Next, any overlapping or sequential duplicates of the same entry were removed and the longest entry in amino acids was used to define the entries location. InterPro entries found in the ORFs are provided [see additional file 4].

Proteins from a subset of genomes (Table 1) were analysed also with Protfun [60] to predict their function and localisation. Protfun categories of proteins are provided [see additional file 5].

### Protein clustering and cluster annotation

Proteins were clustered with the graph clustering software TRIBE-MCL [15]. Briefly, TRIBE-MCL identifies protein family like protein clusters using a Markov Clustering procedure operating on a matrix of expectation values computed from an all-versus-all BLASTP [21] search of protein sequences. TRIBE-MCL requires one major user defined parameter, the inflation value (*r*), which influences the size of the clusters. To define a suitable *r *the InterPro entry structures were used. Each cluster's InterPro entry structure was defined as the most common InterPro entry structure found in its member proteins. Subsequently, for each tested *r *specificity and sensitivity of the clustering (Figure 1) were counted as proposed in [22]. A cluster's specificity was defined as the percentage of proteins in a cluster having the same structure as the cluster's structure. A cluster's sensitivity was defined as the percentage of proteins with the same structure as the cluster in question of all proteins with this structure. Sensitivity and specificity of a clustering were defined as the respective averages of all cluster's sensitivities and specificities in a clustering. Clusters and their members are provided [see additional file 6].

We defined two indexes to describe the difference between clustering with *r *= 1.1 and *r *= 3.1, Stability and "S/P *r *1.1" (Figure 4). The Stability of a cluster is the ratio of cluster sizes between *r *= 3.1 and *r *= 1.1. The cluster size in *r *= 1.1 is the average of the sizes of clusters to which the members of the *r *= 3.1 cluster belong to. "S/P *r *1.1" of a cluster is the ratio between counts of ORFs from Saccharomycotina and Pezizomycotina species. Similarly as Stability, it was counted for an *r *= 3.1 cluster by taking the average of Saccharomycotina – Pezizomycotina ratios of all the clusters to which the members of an *r *= 3.1 cluster belong to in an *r *= 1.1 clustering.

As well as an InterPro entry structure, we assigned individual InterPro entries and Funcat and Protfun categories for clusters to enable analysis of sets of clusters. In addition clusters were checked for presence of proteins found in the *S. cerevisiae *metabolic model iMH805 [25].

### Browsing of protein clusters

To view and browse sequences, their features and protein clusters, a Generic Genome Browser (GBrowse) [13] was set up. Normally GBrowse is used to view individual scaffolds with features i.e. genes. We modified it to show protein sequences with features i.e. domains etc. detected with InterProScan [16] and to display a cluster view where all proteins of a cluster are shown along with their features.

### Data analysis and visualisation

Significance of enrichment of a category in a set of clusters was tested using the hypergeometric distribution with R function "phyper" with default settings i.e. by counting the probability of the number of successes (number of clusters with a certain category) in a sequence of *n *draws from a finite population (total number of clusters, universe) without replacement. Because of the different nature of the cluster annotation data, different universes were used. For Funcat categories all clusters which had any assigned category or entry were used as the universe. For Protfun and Interpro data all clusters having at least 10 members (Figure 4), were used as the universe.

Principal Component Analysis (PCA) was used to find major sources of variation in counts of ORFs having either individual InterPro entries or InterPro entry structures. Counts of ORFs with InterPro entries or InterPro entry structures were normalised with the count of ORFs in a genome. PCA was done with R function "prcomp" with option "retx" set to true.

Heatmaps of ORF counts in clusters or having certain InterPro entries were plotted with modified version of "heatmap.2" from R library "grecmisc". Complete linkage hierarchical clustering with Euclidian distance was used to order rows and columns separately. Prior to clustering one was added to each count, natural logarithm taken and data was mean centred and standard deviation scaled, row or column wise for either clustering respectively.

## Authors' contributions

MA carried out data analysis and drafted the manuscript. TK and MA designed and setup the database and the database browser interface and TK participated in the design of the study. AM carried out InterProScan analysis. MS participated in the design of the study. DU participated in the design of the study. MP and SO conceived the study and participated in the design of the study. All authors have participated in drafting the manuscript and approved the final manuscript.

## Additional files

No data is included for yet unpublished genomes, nor for the *P. pastoris *purchased from ERGO. To find actual sequences search corresponding sequencing centre internet site with the protein sequence identifier.

## Supplementary Material

Additional file 1 Figure 4 as table. An excel version of Figure 4. First sheet contains column title explanations, second sheet the numbers for Figure 4 with cluster identifiers, InterPro structures and Protfun assignments. Last sheet contains Funcat annotations for the clusters in Figure 4. Click here for file

Additional file 2 Figure 9 as table. An excel version of Figure 9. First sheet contains column title explanations, second sheet the numbers for Figure 9 with InterPro identifiers, InterPro names and "Author assingments". Click here for file

Additional file 3 Sequence identifier to species mappings. A text file with species for each protein sequence identifier. First column contains the ORF identifier as provided by the sequencing centre and second column the species. Click here for file

Additional file 4 Sequence indentifier to InterPro identifier mappings. A text file with the Interpro entry identifiers found in each protein sequence. First column contains the ORF identifier as provided by the sequencing centre and second column the identifier for InterPro domain found in the ORF. Click here for file

Additional file 5 Sequence indentifier to ProtFun category mappings. A text file with the Protfun categories found for each protein sequence. First column contains the ORF identifier as provided by the sequencing centre and following columns the ProtFun categories found for the ORF. Click here for file

Additional file 6 Sequence indentifier to cluster identifier mappings. A text file with clusters and their members. First column contains the ORF identifier as provided by the sequencing centre and second column the respective protein cluster. Click here for file
